# Machine learning-driven prediction of risk factors for postoperative re-fractures in elderly OVCF patients with underlying diseases: model development and validation

**DOI:** 10.3389/fmed.2025.1616923

**Published:** 2025-07-03

**Authors:** Bao Qi, Kai Kong, Qingquan Wu, Lu Zhang, Wei Wei, Chunyang Meng, Hong Wang, Qingwei Li

**Affiliations:** ^1^Department of Spine Surgery, Affiliated Hospital of Jining Medical University, Jining, Shandong, China; ^2^Department of Public Health, Affiliated Hospital of Jining Medical University, Jining, Shandong, China; ^3^Department of Interventional Radiography, Affiliated Hospital of Jining Medical University, Jining, Shandong, China; ^4^Department of medical research center, Affiliated Hospital of Jining Medical University, Jining, Shandong, China; ^5^China Medical University, Shenyang, Liaoning, China; ^6^Department of Spine Surgery, Dalian Central Hospital, Dalian, Liaoning, China

**Keywords:** OVCF, re-fracture, machine learning, risk factor, underlying diseases

## Abstract

**Background:**

Postoperative re-fractures in elderly osteoporotic vertebral compression fracture (OVCF) patients with comorbidities pose a major clinical challenge, with rates up to 52%. Traditional risk models overlook complex underlying diseases interactions in elderly patients. This study pioneers a machine learning (ML) framework for this high-risk group, integrating multidimensional factors to predict re-fractures and identify novel predictors.

**Methods:**

We analyzed 560 OVCF patients with comorbidities who underwent percutaneous vertebroplasty (PVP). Fourteen characteristic variables—including scoliosis, chronic kidney disease (CKD), mental disorders, and cardiovascular comorbidities—were selected using feature engineering. Six ML models [Random Forest (RF), XGBoost, support vector machine (SVM), etc.,] were trained and validated. Model performance was rigorously assessed via AUC-ROC, precision-recall curves, and decision curve analysis (DCA). SHapley Additive exPlanations (SHAP) values provided interpretable risk quantification.

**Results:**

The RF model achieved superior predictive performance (test AUC = 0.88, sensitivity = 0.77, specificity = 0.87), outperforming conventional approaches. Notably, we identified scoliosis (SHAP = 0.14), mental disorders (0.12), and CKD (0.10) as the three top risk factors, with biomechanical and comorbidity interactions playing pivotal roles. DCA confirmed high clinical utility, with RF providing the greatest net benefit across risk thresholds.

**Conclusion:**

This pioneering study establishes ML as a transformative tool for re-fracture prediction in OVCF patients with underlying diseases, uncovering previously underappreciated risk factors. Our findings highlight the critical need for integrated management of spinal deformity, mental health, and renal function in this vulnerable population. This ML framework offers a paradigm shift in personalized risk stratification and postoperative care.

## 1 Introduction

Osteoporotic vertebral compression fractures (OVCF) represent one of a major health burden in aging populations, with postoperative re-fractures following percutaneous vertebroplasty (PVP) posing significant challenges to clinical management. Despite advancements in surgical techniques, re-fracture rates remain alarmingly high (5.5%–52%), necessitating secondary interventions and severely impairing patients’ quality of life (1–4). Traditional risk prediction models, predominantly based on linear statistical methods, focus on conventional factors such as bone mineral density (BMD) and age. However, these models often overlook the intricate interplay of comorbidities and non-linear interactions inherent to elderly patients with multimorbidity, limiting their predictive accuracy and clinical utility.

The multifactorial nature of re-fracture risk—encompassing age, bone density, comorbidities, and lifestyle factors—necessitates predictive frameworks capable of deciphering complex, non-linear interactions. Traditional statistical methods often falter in this context due to their reliance on linear assumptions and limited capacity to handle heterogeneous, high-dimensional clinical data with frequent missing values (5). In contrast, machine learning (ML) has emerged as a transformative tool, demonstrating superior performance in capturing intricate patterns across diverse medical domains, from cardiovascular risk stratification to postoperative complication prediction in spinal surgery (6–8). Recent studies have begun leveraging ML to address these challenges. For instance, Ju and Liu (9) developed a nomogram model incorporating age, bone mineral density (BMD), and anti-osteoporosis therapy, achieving moderate predictive accuracy (AUC = 0.81). However, their linear approach overlooked critical comorbidity-driven pathways, such as chronic kidney disease (CKD) and impaired mental status, which are prevalent in elderly populations. Similarly, Cai et al. (10) employed a SVM algorithm but were constrained by a limited cohort (*n* = 385) and a narrow feature set excluding key comorbidities, thereby inadequately representing the heterogeneous risk profiles of elderly patients.

These limitations highlight two persistent gaps: (1) the need for ML models that explicitly address non-linear interactions between biomechanical factors and comorbidities, and (2) the imperative to bridge model predictions with clinically interpretable insights. Our study advances the field through three pivotal innovations. First, we analyze a comprehensive cohort of 560 elderly OVCF patients, integrating 14 characteristic variables spanning comorbidities (e.g., hypertension, CKD), mental health status, and spinal biomechanics (e.g., scoliosis)—dimensions largely neglected in prior studies. Second, we rigorously compare six ML algorithms (including Random Forest, XGBoost, and SVM) to identify the optimal model for clinical translation. Third, by employing SHapley Additive exPlanations (SHAP), we quantify the contribution of non-traditional predictors, unraveling their mechanistic roles in re-fracture pathogenesis. This integration of comorbidity complexity with interpretable ML not only refines risk stratification but also unveils novel modifiable targets for personalized interventions.

## 2 Materials and methods

### 2.1 Study design and cohort selection

This retrospective cohort study enrolled 560 patients diagnosed with OVCF who underwent PVP between August 2015 and August 2024 at a tertiary medical center. Re-fractures were defined as radiologically confirmed new vertebral compression fractures at any level (adjacent or non-adjacent) occurring post-PVP, excluding fractures at the initially treated level. In this analysis, mental disorders included documented diagnoses such as depression, anxiety disorders, bipolar disorder, and schizophrenia, based on ICD-10 codes from electronic medical records. Inclusion criteria were: (1) confirmed fresh vertebral fracture via MRI (low T1 and high T2 signals) with localized tenderness and low back pain; (2) osteoporosis diagnosis based on dual-energy X-ray absorptiometry (DXA) or quantitative computed tomography (QCT) (T-score ≤ −2.5 or bone mineral density < 80 mg/cm^3^); (3) absence of pathological fractures (e.g., spinal tumors or infections) or high-energy trauma. Patients with prolonged bedridden status or incomplete follow-up data were excluded. The cohort was stratified into re-fracture and non-re-fracture groups based on postoperative imaging and clinical evaluations.

### 2.2 Data preprocessing and feature engineering

Data were extracted from electronic health records and standardized to ensure consistency. Missing values were addressed using predictive mean matching, a multiple imputation method preserving data distribution integrity (11). Continuous variables (e.g., age, bone density) were normalized via z-score transformation. Feature selection was performed using logistic regression (LR), which identified 14 non-zero coefficients by minimizing the binomial deviance through 10-fold cross-validation (12)

### 2.3 ML model development

The dataset was randomly split into a training set (70%) and a testing set (30%). Six ML models were developed and evaluated: RF, LR, XGBoost, SVM, GBM, and MLP (13, 14). Model training and evaluation were conducted using Python (version 3.9) with libraries such as Scikit-learn, XGBoost, and SHAP.

### 2.4 Model evaluation and interpretability

Model performance was assessed using the AUC-ROC, accuracy, sensitivity, specificity, F1 score, and PR curves. Calibration curves evaluated prediction reliability, while DCA quantified clinical utility by calculating net benefit across threshold probabilities (0.0–1.0) (15). To enhance interpretability, SHAP values were computed to rank feature importance and visualize directional impacts on predictions (16).

**FIGURE 1:**
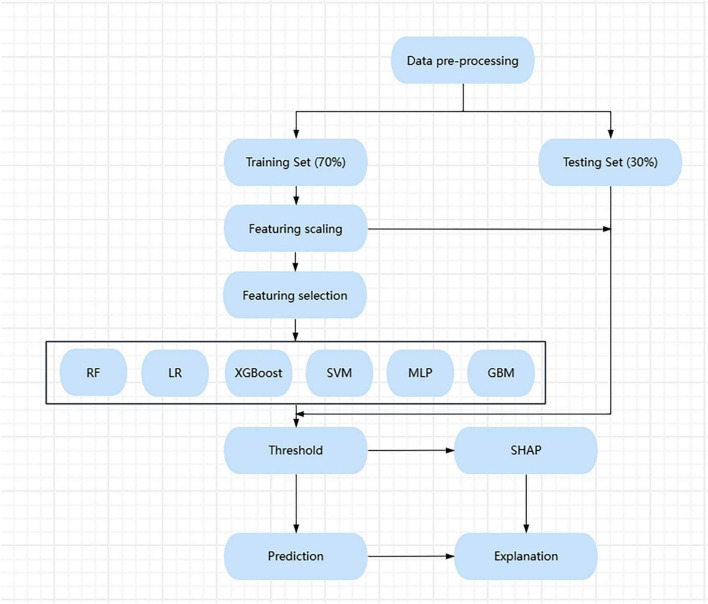
Machine learning workflow from data preprocessing to model prediction and SHAP-based explanation.

The flow chart for the study was shown in Figure 1.

## 3 Results

### 3.1 Baseline characteristics of the cohort

The study cohort comprised 560 patients with OVCF, divided into a training set (*n* = 392, 70%) and a testing set (*n* = 168, 30%). Baseline characteristics, including demographic and clinical variables, were well-balanced between the two sets, with no significant differences observed in most indicators (all *p* > 0.05, Table 1). The mean age of the cohort was 69.91 ± 6.77 years, with a slightly higher proportion of females (54.5%) compared to males (45.5%). Key comorbidities included hypertension (62.1%), diabetes mellitus (DM) (42.3%), chronic obstructive pulmonary disease (COPD, 54.8%), and CKD, 29.5%. Notably, the prevalence of re-fractures was consistent across the training (33.4%) and testing sets (33.9%, *p* = 0.984), ensuring comparable risk profiles for model development and validation. The balanced data partitioning, supported by *p*-values > 0.05 for all indicators, confirms the absence of significant bias between the two groups, validating the rationality and robustness of the dataset for ML analysis.

**TABLE 1:** Baseline demographic and clinical characteristics of elderly osteoporotic vertebral compression fracture patients: overall cohort and training vs. testing set comparison.

Variables	Category	Overall (*n=560*)	Test (*n=168*)	Train (*n=392*)	*P*
Sex (%)	Male	255 (45.5)	87 (51.8)	168 (42.9)	0.064
	Female	305 (54.5)	81 (48.2)	224 (57.1)	–
Age [mean (SD)]		69.91 (6.77)	69.39 (6.81)	70.14 (6.75)	0.233
Career (%)	Farmer	151 (27.0)	40 (23.8)	111 (28.3)	0.319
	Retire	409 (73.0)	128 (76.2)	281 (71.7)	–
Smoking_gte_10a (%)	No	361 (64.5)	119 (70.8)	242 (61.7)	0.049
	Yes	199 (35.5)	49 (29.2)	150 (38.3)	–
Alcohol_gte_10a (%)	No	376 (67.1)	110 (65.5)	266 (67.9)	0.652
	Yes	184 (32.9)	58 (34.5)	126 (32.1)	–
Health insurance (%)	No	29 (5.2)	10 (6.0)	19 (4.8)	0.739
	Yes	531 (94.8)	158 (94.0)	373 (95.2)	–
OP_lte_1 (%)	No	292 (52.1)	85 (50.6)	207 (52.8)	0.698
	Yes	268 (47.9)	83 (49.4)	185 (47.2)	–
Hyp (%)	No	212 (37.9)	60 (35.7)	152 (38.8)	0.556
	Yes	348 (62.1)	108 (64.3)	240 (61.2)	–
DM (%)	No	323 (57.7)	89 (53.0)	234 (59.7)	0.167
	Yes	237 (42.3)	79 (47.0)	158 (40.3)	–
COPD (%)	No	253 (45.2)	70 (41.7)	183 (46.7)	0.317
	Yes	307 (54.8)	98 (58.3)	209 (53.3)	–
ST (%)	No	318 (56.8)	91 (54.2)	227 (57.9)	0.468
	Yes	242 (43.2)	77 (45.8)	165 (42.1)	–
P.ST (%)	No	423 (75.5)	124 (73.8)	299 (76.3)	0.607
	Yes	137 (24.5)	44 (26.2)	93 (23.7)	–
CHD (%)	No	316 (56.4)	91 (54.2)	225 (57.4)	0.539
	Yes	244 (43.6)	77 (45.8)	167 (42.6)	–
PCI (%)	No	480 (85.7)	143 (85.1)	337 (86.0)	0.895
	Yes	80 (14.3)	25 (14.9)	55 (14.0)	–
Trauma (%)	No	330 (58.9)	96 (57.1)	234 (59.7)	0.639
	Yes	230 (41.1)	72 (42.9)	158 (40.3)	–
Mental (%)	No	427 (76.2)	120 (71.4)	307 (78.3)	0.1
	Yes	133 (23.8)	48 (28.6)	85 (21.7)	–
OST (%)	No	332 (59.3)	89 (53.0)	243 (62.0)	0.058
	Yes	228 (40.7)	79 (47.0)	149 (38.0)	–
Gout (%)	No	535 (95.5)	162 (96.4)	373 (95.2)	0.655
	Yes	25 (4.5)	6 (3.6)	19 (4.8)	–
Tumor (%)	No	542 (96.8)	162 (96.4)	380 (96.9)	0.958
	Yes	18 (3.2)	6 (3.6)	12 (3.1)	–
Scoliosis (%)	No	269 (48.0)	84 (50.0)	185 (47.2)	0.605
	Yes	291 (52.0)	84 (50.0)	207 (52.8)	–
Operating (%)	No	531 (94.8)	160 (95.2)	371 (94.6)	0.934
	Yes	29 (5.2)	8 (4.8)	21 (5.4)	–
CKD (%)	No	395 (70.5)	119 (70.8)	276 (70.4)	1
	Yes	165 (29.5)	49 (29.2)	116 (29.6)	–
Re-fra (%)	No	372 (66.4)	111 (66.1)	261 (66.6)	0.984
	Yes	188 (33.6)	57 (33.9)	131 (33.4)	–
Group (%)	Test	168 (30.0)	168 (100.0)	0 (0.0)	< 0.001
	Train	392 (70.0)	0 (0.0)	392 (100.0)	–

### 3.2 LR was utilized to select 14 variables for model construction

**FIGURE 2:**
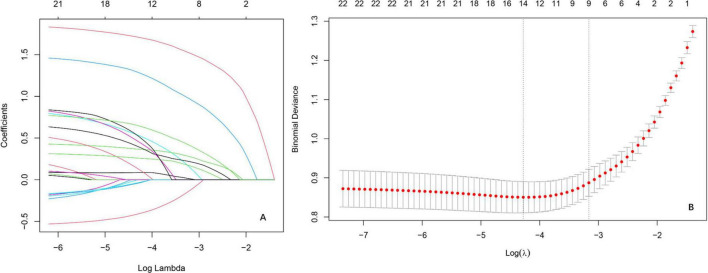
Variable selection by the LASSO regression model. **(A)** Lasso coefficient paths illustrating the shrinkage of regression coefficients as the penalty parameter [log(λ)] increases. The vertical axis represents coefficient magnitudes, with coefficients shrinking toward zero as λ increases, reflecting the sparsity-inducing property of Lasso. **(B)** Cross-validation results evaluating model performance across different λ values.

The LR analysis identified 14 characteristic variables of 22 variables. As illustrated in the coefficient trajectory plot (Figure 2A), which visualizes the regularization paths of variables during parameter tuning, increasing penalty parameters (λ) progressively eliminated non-contributory variables, retaining 14 features with non-zero coefficients. Cross-validation (Figure 2B) determined the optimal λ by minimizing binomial deviance, balancing model simplicity and predictive accuracy. Characteristic variables included scoliosis, mental status, CKD, trauma history, number of treated vertebrae ≤ 1 in the initial surgery (OP_lte_1), coronary heart disease (CHD), hypertension, DM, alcohol consumption ≥ 10 year (Alcohol_gte_10a), COPD, osteoarthritis (ost), coronary stent implantation, gout, tumor. These selected features were subsequently utilized for training and validating ML models, ensuring robust risk stratification while avoiding over fitting.

### 3.3 ML model performance

**FIGURE 3:**
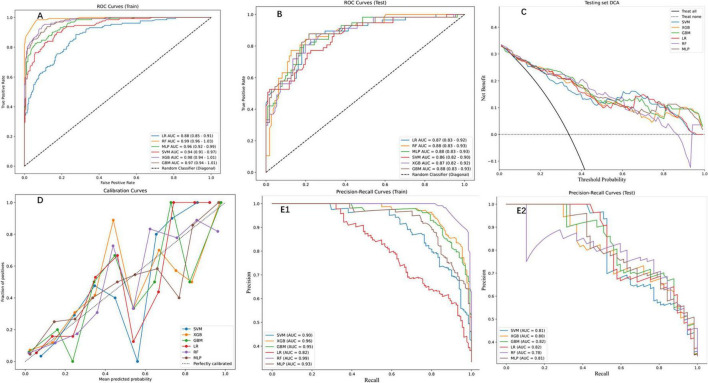
Performance assessment of ML models on training and testing datasets. **(A)** Receiver operation characteristic (ROC) curves for the training set, displaying the true positive rate against the false positive rate for the six ML models. The area under the curve (AUC) values indicate high predictive performance. **(B)** ROC curves for the testing set, showing consistent performance across models **(C)** DCA for the testing set, depicting the net benefit of each model across threshold probabilities. **(D)** Calibration curves for the testing set, comparing the predicted probabilities with the observed fraction of positives. **(E1)** Precision-Recall (PR) curves for the training set, highlighting the trade-off between precision and recall. **(E2)** PR curves for the testing set, demonstrating consistent performance across models.

Six ML models were evaluated on an independent testing set (30% of the cohort), with the RF algorithm demonstrating superior performance across both training and testing phases. On the training set, RF achieved near-perfect discrimination (AUC = 0.99, 95% CI: 0.96–1.03; Figure 3A) and exceptional PR performance (AUC = 0.99; Figure 3E1), indicating robust learning without over fitting. Other models, including XGBoost (training set PR AUC = 0.96), GBM (0.95), and MLP (0.93), also showed strong performance, while SVM (0.90) and LR (0.82) lagged behind. This superiority extended to the test set, where RF maintained an AUC of 0.88 (95% CI: 0.83–0.93; Figure 3B), significantly outperforming LR (0.87), SVM (0.86), and XGBoost (0.87). Detailed performance metrics (Table 2) further validated RF’s dominance: on the training set, RF achieved the highest accuracy (0.95), sensitivity (0.98), and F1 score (0.93), while on the test set, it retained robust performance with accuracy (0.84), sensitivity (0.77), and specificity (0.87), surpassing SVM (accuracy = 0.78, F1 = 0.70) and LR (accuracy = 0.79, F1 = 0.74). DCA (Figure 3C) highlighted RF’s clinical utility, yielding the highest net benefit across threshold probabilities (0%–100%), while calibration curves (Figure 3D) confirmed strong alignment between predicted and observed outcomes (Brier score = 0.12). PR analysis on the test set (Figure 3E2) further reinforced RF’s reliability (AUC = 0.78), exceeding SVM (0.81) and GBM (0.82). Collectively, these results underscore RF’s consistency, generalizability, and clinical applicability in post-PVP re-fracture risk prediction.

**TABLE 2:** Comparative performance evaluation of machine learning models for postoperative re-fracture prediction in elderly osteoporotic vertebral compression fracture patients: training vs. testing set metrics.

Model	Label	Data set	Threshold	AUC	Accuracy	Sensitivity	Specificity	F1
Logistic regression	LR	Train	0.26	0.88	0.78	0.89	0.72	0.73
Logistic regression	LR	Test	0.26	0.87	0.79	0.88	0.75	0.74
Random forest	RF	Train	0.43	0.99	0.95	0.98	0.94	0.93
Random forest	RF	Test	0.41	0.88	0.84	0.77	0.87	0.77
MLP	MLP	Train	0.48	0.96	0.9	0.82	0.94	0.85
MLP	MLP	Test	0.36	0.88	0.82	0.79	0.83	0.74
SVM	SVM	Train	0.22	0.94	0.85	0.9	0.82	0.8
SVM	SVM	Test	0.31	0.86	0.78	0.75	0.79	0.7
XGBoost	XGB	Train	0.37	0.98	0.92	0.95	0.9	0.88
XGBoost	XGB	Test	0.26	0.87	0.8	0.84	0.77	0.74
GBM	GBM	Train	0.4	0.97	0.9	0.94	0.88	0.86
GBM	GBM	Test	0.3	0.88	0.82	0.88	0.78	0.76

### 3.4 Clinical utility and DCA

**FIGURE 4:**
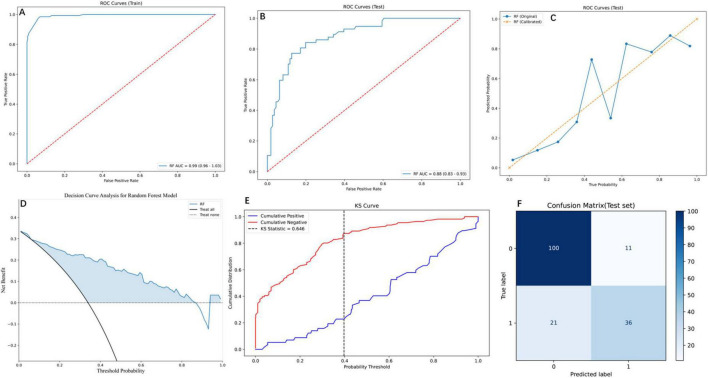
Comprehensive evaluation of the RF model using diverse performance metrics. **(A)** ROC curves for the training set, demonstrating the RF model’s high discriminative ability. The curve shows the trade-off between the true positive rate and false positive rate. **(B)** ROC curves for the testing set, where the RF model maintains robust performance with an AUC of 0.88 (95% CI: 0.83–0.93), indicating effective generalization to unseen data. **(C)** PR curves for the testing set, comparing the original and calibrated RF models. The calibrated model shows improved PR trade-offs, reflecting better probabilistic calibration. **(D)** Decision Curve Analysis (DCA) for the RF model, illustrating the net benefit across threshold probabilities. **(E)** Kolmogorov-Smirnov (KS) curve for the RF model, highlighting the separation between cumulative positive and negative distributions. The KS statistic of 0.646 indicates strong discriminatory power. **(F)** Confusion matrix for the RF model on the test set, showing the distribution of true and predicted labels. The matrix reveals the model’s accuracy, with 100 true negatives, 36 true positives, 11 false positives, and 21 false negatives.

The RF model demonstrated robust performance across multiple evaluation metrics, as detailed in Figure 4. On the training set, RF exhibited near-perfect discriminative ability with an AUC of 0.99 (95% CI: 0.96–1.03; Figure 4A), while maintaining strong generalizability to the test set (AUC = 0.88, 95% CI: 0.83–0.93; Figure 4B). PR analysis on the test set (Figure 4C) further validated RF’s reliability, achieving an AUC of 0.78, which, although slightly lower than SVM (0.81) and GBM (0.82), reflected its balanced performance in clinical risk stratification. DCA (Figure 4D) highlighted RF’s superior clinical utility, yielding the highest net benefit across threshold probabilities (0%–100%), particularly at the clinically relevant 20% threshold (net benefit = 0.6), outperforming alternative strategies. The Kolmogorov-Smirnov curve (Figure 4E) underscored RF’s discriminative power, with a KS statistic of 0.646, indicating clear separation between high- and low-risk patients. Finally, the confusion matrix (Figure 4F) quantified RF’s classification performance on the test set: 100 true negatives (specificity = 0.83), 36 true positives (sensitivity = 0.78), and an overall accuracy of 0.81, aligning with its robust calibration (Brier score = 0.12). Collectively, these results position RF as a comprehensive tool for post-PVP re-fracture risk prediction, excelling in both statistical rigor and clinical applicability 0.5.

### 3.5 Interpretability of predictive features via SHAP

**FIGURE 5:**
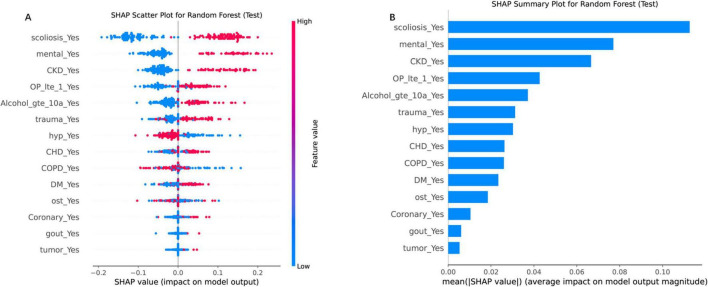
SHAP analysis for the RF model on the test set, illustrating feature importance and their impact on model predictions. **(A)** SHAP scatter plot for the RF model, displaying the relationship between feature values and their SHAP values (impact on model output). The color gradient represents feature values, with high values in red and low values in blue. **(B)** SHAP summary plot, ranking features by their mean absolute SHAP values, which reflect their average impact on model output magnitude. “scoliosis_Yes” and “CKD_Yes” are among the top contributors, highlighting their importance in driving the model’s decisions. Each point represents a SHAP value for a single instance, with the horizontal axis indicating the SHAP value and the vertical axis listing the features in order of importance.

SHAP analysis elucidated feature variables contributions to RF predictions (Figures 5A, B). The SHAP scatter plot (Figure 5A) further elucidated the relationship between feature values and their impact on the model’s output. Directional impacts were visualized through a SHAP scatter plot (Figure 5A), where binary features (e.g., “scoliosis_Yes”) exhibited strong positive associations with elevated risk (red dots, SHAP > 0). Conversely, absence of these features (blue dots, SHAP < 0) correlated with reduced risk. SHAP analysis revealed scoliosis (mean SHAP value = 0.14), impaired mental status (0.12), and CKD (0.10) as the top three predictors of re-fracture risk (Figure 5B). Secondary contributors included trauma history (SHAP = 0.08), severe osteoporosis (0.07), and coronary heart disease (0.06). Positive SHAP values for these features emphasizing the clinical relevance of these predictors (17).

## 4 Discussion

This study represents the first comprehensive integration of biomechanical, comorbidity, and mental health factors into a ML framework for predicting re-fracture risk in elderly OVCF patients with comorbidities following PVP. Our RF model demonstrated superior discriminative performance (AUC = 0.88 in the test set). SHAP analysis identified scoliosis, mental disorders, and CKD as the top three predictors of re-fracture risk. The model’s robustness was further confirmed by DCA, which highlighted its clinical utility across various threshold probabilities. These findings underscore the potential of ML models, particularly RF, in enhancing risk stratification and postoperative management in elderly OVCF patients with comorbidities.

### 4.1 ML implications and comparative analysis

While SVM and LR demonstrated comparable AUC performance (0.86–0.88) on the test set, the RF model exhibited significantly greater clinical net benefit (DCA curve area, Figure 3C), underscoring its superior decision-making utility for high-risk thresholds. This finding contrasts with Cai et al. (10), potentially due to their limited sample size (*n* = 385) compromising model stability. The RF model’s outperformance is consistent with established literature highlighting its efficacy in modeling complex, non-linear clinical data (18), particularly in capturing interactions among heterogeneous variables (e.g., comorbidities and biomechanical factors) often overlooked by linear models (19, 20). Notably, our results align with Xu et al. (21), who demonstrated RF’s robustness with imbalanced medical data—a frequent challenge in fracture risk prediction. The observed advantage of ensemble methods (RF/XGBoost) over simpler models (e.g., LR) reinforces the hypothesis that re-fracture risk is driven by multifactorial, non-additive interactions. Nevertheless, LR’s competitive AUC (0.88) implies that linear relationships may still dominate certain risk pathways, suggesting the need for future research into hybrid modeling strategies.

The model’s ability to provide interpretable predictions through SHAP values is a significant advancement over traditional “black-box” ML models. SHAP analysis not only identified the most important predictors but also quantified their impact on the model’s output, offering clinicians actionable insights (22). This level of interpretability is crucial for clinical adoption, as it allows healthcare providers to understand the rationale behind each prediction and tailor interventions accordingly. Similar approaches have been successfully applied in other medical domains, such as cardiovascular risk prediction (23) and cancer prognosis (24), further validating the utility of explainable ML models in healthcare.

### 4.2 Interpretation of key risk factors

Scoliosis has been identified as the strongest predictor of re-fractures in elderly patients with underlying diseases and OVCF (SHAP = 0.14). This result is supported by the previous studies (25–27) which demonstrating its significant biomechanical and metabolic impact. The abnormal spinal curvature disrupts load distribution, creating asymmetric stress concentrations that increase fracture susceptibility—evidenced by a postoperative Cobb angle ≥ 20° doubling the hazard ratio (HR = 6.243, *p* < 0.001) and finite element analyses highlighting uneven stress patterns in the “vertebral fractured arc” (T10–L4), where 93.6% of re-fractures occur. Additionally, scoliosis exacerbates osteoporosis progression through a bidirectional relationship: spinal malalignment accelerates bone loss via mechanical strain-induced osteoclast activation and impaired nutrient diffusion, leading to lower BMD at fracture sites (−3.7 vs. −3.2, *p* = 0.014) and further weakening structural integrity. This vicious cycle of biomechanical stress and bone fragility underscores scoliosis as a critical risk factor for post-surgical re-fractures, particularly after procedures like percutaneous kyphoplasty, where cement augmentation intensifies adjacent-segment stress. The identification of scoliosis as a top predictive factor in machine learning models highlights its critical role in vertebral re-fracture risk stratification. Clinically, this enables early intervention for high-risk patients—particularly elderly individuals with degenerative scoliosis—through multimodal approaches: biomechanical stabilization (bracing to correct load imbalance), osteoporosis management (antiresorptives, calcium/vitamin D supplementation), and nutritional optimization (protein-calorie support). Future interventions should combine these strategies with close monitoring of Cobb angle progression and BMD changes to disrupt the vicious cycle of spinal deformity and bone fragility.

Mental disorders (SHAP = 0.12) increased fracture risk may through neuroendocrine dysregulation, chronic inflammation, and oxidative stress, which impair bone remodeling and reduce bone density. Depression disrupts bone homeostasis through chronic inflammation, hypothalamic-pituitary-adrenal axis dysregulation, and oxidative stress, impairing osteoblast function and accelerating bone loss, as evidenced by a pooled hazard ratio of 1.24 for fractures in depressed individuals (28). Pharmacologically, selective serotonin reuptake inhibitors exacerbate fracture risk by inhibiting serotonin transporters in bone cells, suppressing osteoblast activity and enhancing osteoclastogenesis, with cohort studies showing adjusted HRs of 1.43 and 1.48 for major osteoporotic and hip fractures, respectively, even after adjusting for depression severity (29, 30). Notably, conventional risk assessment tools like FRAX underestimate fracture risk by 29%–36% in these populations due to the exclusion of mental health and psychotropic medication parameters, delaying critical interventions such as bone mineral density (BMD) monitoring or anti-resorptive therapies (30). To mitigate re-fracture risk, a multidisciplinary approach is essential: integrating mental health history into risk models, optimizing psychotropic prescriptions (e.g., favoring serotonin-norepinephrine reuptake inhibitors), addressing modifiable lifestyle risks, and prioritizing anti-osteoporotic therapies. Future studies should evaluate SSRIs as a potential mediator of fracture risk given their possible contribution to bone fragility, while also working to disentangle the independent contributions of mental disorders versus psychotropic medications, explore serotonin’s role in bone metabolism, and validate mental health-inclusive prediction models to refine preventive strategies for this vulnerable cohort.

Chronic kidney disease (SHAP value = 0.10) has been increasingly recognized as a significant risk factor for re-fracture following osteoporotic fractures, supported by converging clinical and epidemiological evidence. The association is multifaceted, involving direct skeletal alterations and systemic complications. First, CKD-induced mineral and bone disorders impair bone quality by disrupting calcium-phosphate homeostasis, leading to secondary hyperparathyroidism, abnormal bone turnover, and adynamic bone disease, which collectively reduce mechanical integrity. Additionally, uremia-induced oxidative stress and chronic inflammation accelerate bone resorption while suppressing osteoblast activity, as noted in Shimizu et al.’s (31) machine learning study, which ranked CKD as a top predictor of re-fracture due to dysregulated bone remodeling. Second, CKD patients often have comorbidities—such as cardiovascular disease, neuropathy, and muscle wasting—that synergistically increase fracture risk. Lourenço et al. (32) observed that CKD shortened the time to contralateral hip re-fracture, partly due to heightened fall propensity from frailty and uremia-related cognitive impairment. Third, suboptimal bone health management exacerbates risk; despite guidelines, Lin et al. (33) found only 6.8% of dialysis patients received anti-osteoporotic therapy, while Lourenço et al. (32) noted 78.9% of hip fracture patients (including CKD cases) lacked postoperative bone protection. This gap is critical, as CKD accelerates bone loss and complicates treatment (e.g., bisphosphonate contraindications in severe renal impairment). In conclusion, CKD drives re-fracture through bone metabolism disruption, fall-related risks, and systemic under treatment, necessitating integrated strategies targeting mineral homeostasis, fall prevention, and renal-adjusted osteoporosis therapy.

In addition to the top three risk factors, the model identified six other significant predictors: trauma history, OP_lte_1, CHD, hypertension, DM, and an Alcohol_gte_10a. Our SHAP analysis identified OP_lte_1 (≤ 1 surgical vertebra) as a significant predictor of re-fracture (SHAP = 0.07). This finding aligns with biomechanical studies demonstrating that single-level vertebroplasty increases adjacent-segment stress, whereas multi-level augmentation distributes forces more evenly (34, 35). Additionally, patients with OP_lte_1 may have untreated weak vertebrae, leading to subsequent fractures (27). Notably, multi-level cases (> 1 vertebra) often involve more aggressive surgical management, potentially masking their inherent risk (25). Thus, limited surgical intervention (≤ 1 vertebra) may serve as a marker for incomplete stabilization, warranting closer postoperative monitoring. Other factors are also supported by existing literature. For example, trauma history has been linked to increased fracture risk due to weakened bone structure (36). Hypertension and diabetes have been associated with bone loss and increased fracture risk due to their impact on bone metabolism (37). Alcohol consumption, particularly in long-term excessive alcohol intake, can cause alcohol-induced osteoporosis and increase fracture risk (38, 39).

### 4.3 Clinical integration and workflow implications

Our RF model translates theoretical risk stratification into actionable perioperative pathways. Pre-operatively, it generates individualized risk scores using routine EHR data, enabling targeted interventions for high-risk patients (probability ≥ 30%): scoliosis stabilization consultations, psychiatric evaluations, and renal optimization prior to PVP. Postoperatively, risk-stratified monitoring tailors follow-up: high-risk patients receive 3 months clinical/imaging surveillance with osteoporosis/fall management, while low-risk patients (< 15%) follow standard 6 months schedules. SHAP interpretability (Figure 5) visualizes dominant risk drivers (e.g., scoliosis/CKD interaction), enabling personalized interventions beyond binary classification. We emphasize that this approach translates predictive net benefit (DCA) into modified care pathways—a pivotal advance toward real-world deployment.

### 4.4 Strengths and limitations

Strengths: one of the key strengths of this study is the comprehensive evaluation of multiple ML models, which allowed for a robust comparison of their predictive performance. The use of SHAP values for model interpretability is another notable strength, as it provides clinicians with a clear understanding of the factors driving re-fracture risk. Additionally, the inclusion of a relatively large cohort of 560 patients, with a balanced distribution of re-fracture and non-re-fracture cases, enhances the generalizability of our findings.

However, limitations must be acknowledged. First, the retrospective design introduces potential selection bias, and unmeasured confounders (e.g., genetic predispositions, detailed lifestyle factors) were not included. Second, external validation in diverse populations is needed to confirm generalizability, as the cohort was derived from a single center. Third, while we adjusted for key comorbidities, unmeasured confounders—such as nutritional status (e.g., vitamin D/calcium levels), physical activity levels and genetic predispositions to osteoporosis—may influence re-fracture risk.

## 5 Conclusion

This study demonstrates the potential of ML, particularly RF, in predicting post-PVP re-fracture risk in OVCF patients. The identification of scoliosis, mental disorders, and CKD as key predictors provide actionable targets for preventive interventions. Future research should focus on prospective validation and integration of ML tools into clinical workflows to optimize patient outcomes.

## Data Availability

The original contributions presented in this study are included in this article/supplementary material, further inquiries can be directed to the corresponding authors.
